# **AstuteClinician Report: A Novel 10 bp Frameshift Deletion in Exon 2 of*ICOS*Causes a Combined Immunodeficiency Associated with an Enteritis and Hepatitis**

**DOI:** 10.1007/s10875-015-0193-x

**Published:** 2015-09-23

**Authors:** Nic Robertson, Karin R. Engelhardt, Neil V. Morgan, Dawn Barge, Andrew J. Cant, Stephen M. Hughes, Mario Abinun, Yaobo Xu, Mauro Santibanez Koref, Peter D. Arkwright, Sophie Hambleton

**Affiliations:** Primary Immunodeficiency Group, Institute of Cellular Medicine, Newcastle University, Newcastle upon Tyne, NE2 4HH UK; Great North Children’s Hospital, Newcastle upon Tyne Hospitals NHS Foundation Trust, Newcastle upon Tyne, UK; Centre for Cardiovascular Sciences, School of Clinical and Experimental Medicine, University of Birmingham, Birmingham, UK; Blood Sciences Flow Cytometry Laboratory, Newcastle upon Tyne Hospitals NHS Foundation Trust, Newcastle upon Tyne, UK; Paediatric Immunology, Royal Manchester Children’s Hospital, Manchester, UK; Institute of Genetic Medicine, Newcastle University, Newcastle upon Tyne, UK

**Keywords:** ICOS, primary immunodeficiency, common variable immunodeficiency, CVID

## Abstract

*ICOS* encodes the Inducible T-cell Co-Stimulator (ICOS). Deficiency of this receptor in humans causes a common variable immunodeficiency (CVID) characterised by an absence of class-switched memory B cells and hypogammaglobulinemia. Three pathogenic mutations in *ICOS* have been described to date in a total of 13 cases. Here we report a novel homozygous 10 base pair frameshift deletion in exon 2 discovered by whole exome sequencing of two siblings from a family of Pakistani origin. Both patients presented in early childhood with diarrhea, colitis and transaminitis and one showed defective handling of human herpesvirus 6. Activated patient CD3^+^CD4^+^ T lymphocytes demonstrated a complete absence of ICOS expression and, consistent with previous reports, we detected a reduction in circulating T follicular helper cells. Findings in this kindred emphasise the phenotypic variability of ICOS deficiency and, in particular, the variably impaired antiviral immunity that is a poorly understood facet of this rare disorder.

**Electronic supplementary material:**

The online version of this article (doi:10.1007/s10875-015-0193-x) contains supplementary material, which is available to authorized users.

## Introduction

The Inducible T-cell Co-Stimulator (ICOS) is a receptor structurally related to CD28. ICOS is expressed by T cells following activation while its ligand (ICOS-L) is expressed on antigen presenting cells including B cells [1]. Co-stimulation through ICOS enhances many aspects of helper T cell function and is important in the generation of multiple lymphocyte subsets; currently its most clearly defined role in humans is in the differentiation of T follicular helper cells (Tfh) [2–4].

A homozygous 1815 base-pair deletion in the ICOS-encoding gene *ICOS* was reported in 2003 as the cause of adult-onset autosomal recessive common variable immunodeficiency in multiple patients from the Danube region [5]. Subsequently, two different pathogenic single base-pair frameshift deletions in exon 2 have been identified in homozygosity, the first in two siblings from Japan and the second, most recently, in two siblings from Kuwait [6, 7]. The phenotypic spectrum of ICOS deficiency has expanded as more patients are identified, with features varying from refractory diarrhea in early life to adult onset infection, autoimmunity and neoplasia [7–9].

We investigated two siblings who presented in early childhood with persistent pathogen-negative diarrhea and identified a novel homozygous 10 base-pair deletion in exon 2 of *ICOS*.

## Case Overview

**Fig. 1:**
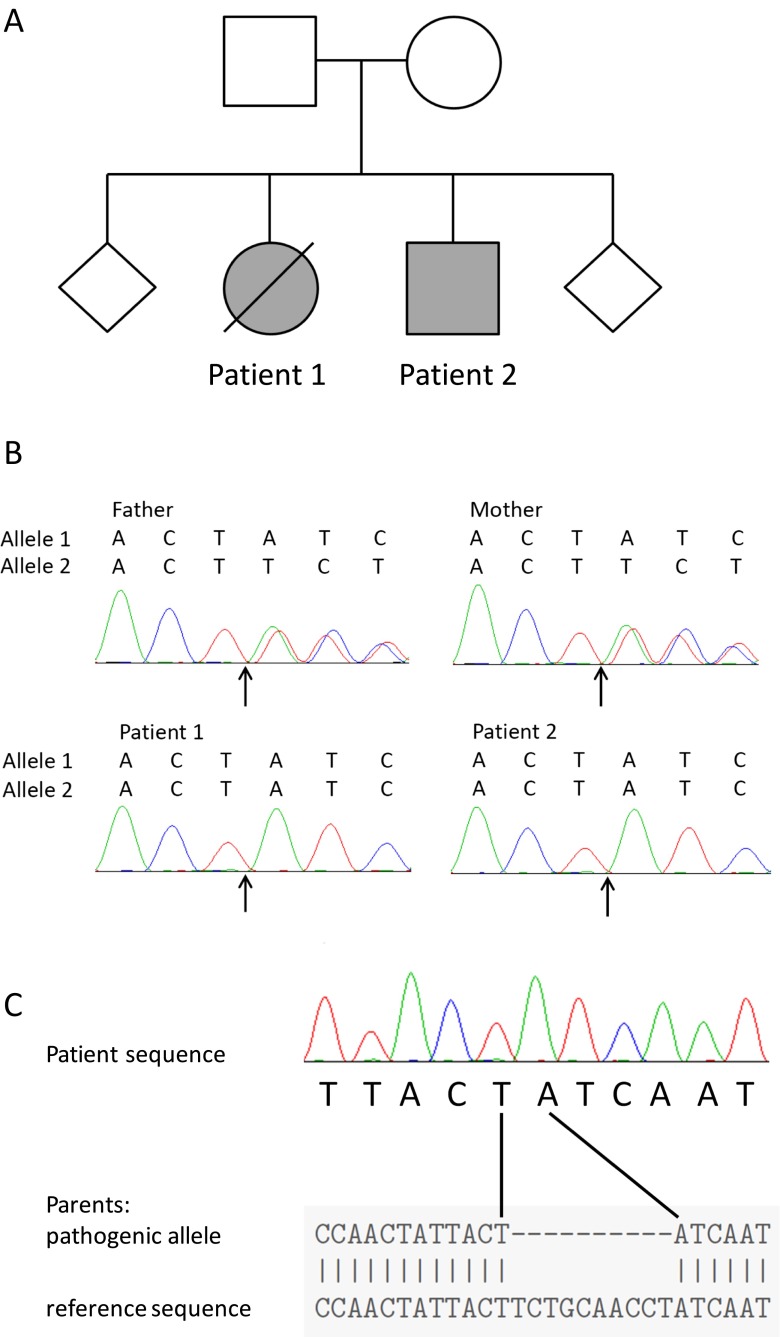
10 base pair deletion in *ICOS* in two siblings. **a** Family tree. Diamonds represent healthy siblings whose gender is not disclosed to protect the family’s privacy. **b** Sanger sequencing of family members. Arrow indicates the start of the deletion. **c** Alignment of patient sequence with a parental sequence reconstructed by Poly Peak Parser to show reference and pathogenic alleles [10]

The parents of the patients are from the same ethnic background and are not, to their knowledge, related (Fig. 1a). From the age of 2 years, their second child (patient 1) suffered from chronic loose watery stools associated with abdominal pain, fever, lethargy and weight loss. She was referred to immunology services aged 3.5 years and was noted to have absent class-switched memory B-cells (CD19 + CD27 + IgD-), hypogammaglobulinemia and impaired vaccine responses (Table 1). She was commenced on immunoglobulin replacement therapy but diarrhea persisted and she developed hepatomegaly associated with raised liver enzymes (alanine aminotransferase [ALT] of 2907 IU/L, normal range 10 to 40; gamma-glutamyl transferase [GGT] 152 IU/L, normal range 0–51). Stools were free of enteric pathogens with the exception of single samples positive for norovirus, adenovirus and *Cryptosporidium*.

**Table 1:** Immunological parameters from ICOS deficient patients

Parameter	Patient 1	Patient 2	Reference range [11–14]
Neutrophils	4.7 × 10^9^/L	5.82 × 10^9^/L	1.5–8 × 10^9^/L
Lymphocytes	2.9 × 10^9^/L	5.3 × 10^9^/L	1.7–6.9 × 10^9^/L
CD3+	2352 cells/μl	2856 cells/μl	900–4500 cells/μl
CD4+	1748 cells/μl	1877 cells/μl	500–2400 cells/μl
CD8+	469 cells/μl	879 cells/μl	300–1600 cells/μl
CD19+	1607 cells/μl	2824 cells/μl	200–2100 cells/μl
CD19 + CD27-IgD+ (naïve B Cell)	86 %	97 %	83·4–90·1 %
CD19 + CD27 + IgD+ (memory B cell)	2 %	3 %	4·2–6·9 %
CD19 + CD27 + IgD- (class switched B Cell)	<1 %	0 %	1·5–4·1 %
CD4-CD45RA + CD27- (effector CD8+)	0 cells/μl	86 cells/μl	
CD4-CD45RA + CD27+ (naïve CD8+)	353 cells/μl	742 cells/μl	
CD4 + CD45RA + CD27+ (naïve CD4+)	706 cells/μl	799 cells/μl	
Activated T cells (HLA-DR+)	5 %	7 %	
IgM	0.17 g/L	0.32 g/L	0.48–1.68 g/L
IgG	2.07 g/L	1.5 g/L	4.24–10.51 g/L
IgA	0.43 g/L	0.29 g/L	0.14–1.23 g/L
Tetanus	non-protective	non-protective	
Hib	0.36 ųg/ml	0.02 ųg/ml	0.22–42.8 ųg/ml
Pneumococcal serotypes: protective responses	0/12 serotypes	2/12 serotypes	
Measles IgG	Not done	Positive	
Mumps IgG	Not done	Positive	
Rubella IgG	Not done	Negative	

Liver and gut biopsies showed mild chronic hepatitis and severe active chronic panenteritis, respectively. Samples from sigmoid colon, duodenum and liver were PCR positive for human herpesvirus 6 (HHV6). Treatment with intravenous ganciclovir and then oral valganciclovir together with nitazoxanide resulted in some improvement of the hepatitis: liver and duodenal samples became negative for HHV6 but sigmoid biopsy remained positive. However, the patient continued to have clinically severe colitis with persistent diarrhea and abdominal pain which limited her school attendance. By analogy with other combined immunodeficiencies we were concerned that this situation would only deteriorate over time. Following extensive discussion among the team and with family, it was therefore decided to attempt curative treatment with hematopoietic stem cell transplantation (HSCT). She received an unrelated 11/12 HLA matched transplant following reduced intensity conditioning. Unfortunately she developed capillary leak syndrome on day 5 with respiratory distress followed by toxic epidermal necrolysis and died from these complications.

The younger brother of the proband (patient 2) presented similarly at age two years with an episode of diarrhea. Investigations again revealed low immunoglobulins, absent class-switched memory B-cells and raised liver enzymes (Table 1 and data not shown). PCR analyses of blood for CMV, EBV, HHV6 and adenovirus were negative. The family have so far refused immunoglobulin replacement therapy; the patient is 7 years old and currently remains well.

## Investigations

We explored the hypothesis that the affected siblings had an autosomal recessive disorder caused by homozygosity for a mutation derived from a shared parental ancestor. DNA from both children was submitted for whole exome sequencing and analysed in conjunction with homozygosity mapping data (full methods in supplemental file). Filtering out common variants left a homozygous 10 base-pair deletion in *ICOS* as the only plausible candidate disease-causing variant in the linkage regions. Sanger sequencing confirmed segregation in keeping with autosomal recessive inheritance: both parents possessed one wild-type allele and one allele carrying the deletion (c.321_330del; Fig. 1b and c), while both affected children were homozygous for the deletion. The deletion leads to a frameshift and a premature stop after 10 codons in the new reading frame (p.F108YfsX118).

**Fig. 2:**
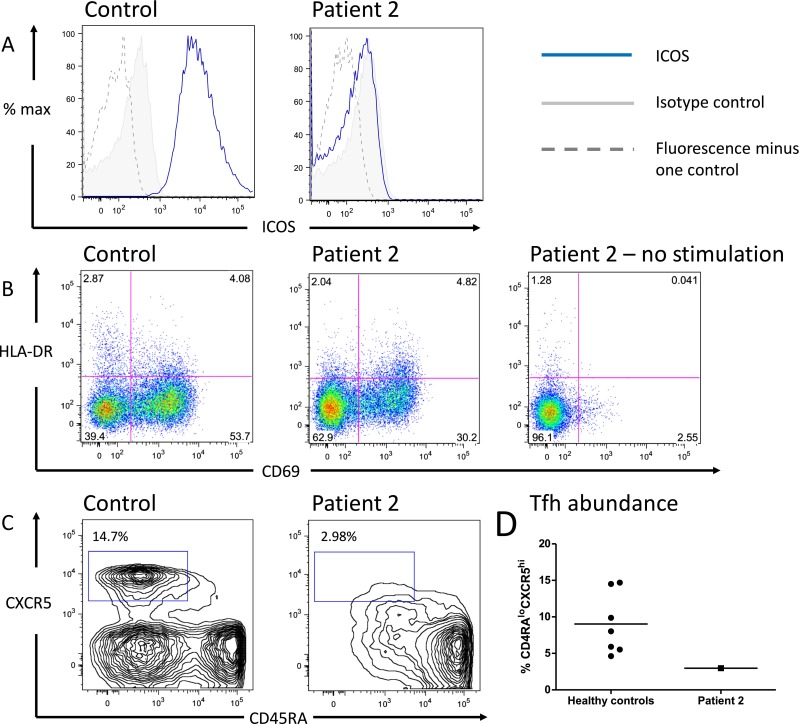
Frameshift deletion in *ICOS* causes failure of ICOS expression on activated T cells and reduced Tfh. **a** ICOS expression on control and patient cells. Peripheral blood mononuclear cells (PBMCs) were stimulated with PHA for 18 h before staining. Plots are gated on live CD3^+^CD4^+^ lymphocytes. **b** ICOS deficiency does not impair T cell activation as assessed by CD69 expression. Cells were treated and gated as in (A). **c** Reduction in circulating Tfh in ICOS deficient patient. Healthy control or patient PBMCs were stained for the Tfh markers CXCR5 and CD45RA. Plots are gated on live CD3^+^CD4^+^ lymphocytes. **d** Quantification of experiment shown in C showing multiple healthy controls. Line indicates mean value

To confirm ICOS deficiency at protein level, cryopreserved frozen aliquots of peripheral blood mononuclear cells from patient 2 were analyzed by flow cytometry after stimulation with PHA. This demonstrated complete absence of ICOS expression (Fig. 2a) despite upregulation of the T-cell activation marker CD69 (Fig. 2b). Unfortunately no cryopreserved material for this assay was available from patient 1.

Previous reports have concluded that there is an association between ICOS deficiency and a reduction in the circulating pool of Tfh, as represented by the CXCR5^hi^ proportion of the memory (CD4RA^lo^) peripheral CD4^+^ T cell population [7, 15]. Defective Tfh generation would be consistent with the histopathological finding of aberrant germinal centres in ICOS deficient patients [9, 16]. In contrast to all controls tested, patient 2 had no discrete population of CD45RA^lo^CXCR5^hi^CD4^+^ T cells, and the overall proportion of CD4^+^ lying in this region was lower than the normal range (2.98 % of CD4^+^ cells compared to an average of 9.02 % in controls, range 4.62–14.70 %; Fig. 2d). Patient 2 had a relatively low total proportion of CD45RA^−^ helper memory T cells at 22.4 % of the total CD3^+^CD4^+^ lymphocyte population. However, this figure lies within the normal range both from our adult controls (where the figure was 18.7–68.1 %), and a previously reported reference range for the patient’s age group (approximately 15–65 %) [17].

## Discussion

This report clearly demonstrates that ICOS-deficiency can be associated with clinical features of cellular as well as humoral immunodeficiency. The most common presentation in previous cases was pneumonia, which could be mechanistically explained by defective antibody production [9]. In contrast, features described here and in the recently reported Kuwaiti siblings suggest a broader disorder of T cell function: patient 1 demonstrated defective handling of HHV6 and possibly also of *Crytosporidium*, while the Kuwaiti patients suffered from *Pneumocystis jirovecii* pneumonia (PJP) and cytomegalovirus viremia [7]. Thus the clinical features of ICOS deficiency parallel those of another T-B cell costimulatory defect, CD40 ligand deficiency, in which patients not only show hypogammaglobulinemia but also evidence of an accompanying T-cell immunodeficiency, commonly in the form of *Pneumocystis* or *Cryptosporidium* infections [18].

Although a less prominent feature, earlier reports of ICOS deficiency also included evidence of impaired viral immunity. Patients from the Danube cohort have been reported to experience recurrent HSV infection and one individual developed HPV-driven vulval carcinoma, while one of the Japanese siblings is described as having prolonged viral infections in infancy [6, 9]. The mechanism behind this dysfunction is not entirely clear but may be related to defective cytokine secretion [1]. One factor in the milder course of our patient 2 to date may relate to age of exposure to microbial pathogens: age at presentation varies widely in both ICOS and CD40 ligand deficiency, but CD40 ligand deficiency appears to be more consistently apparent in childhood [9, 18].

Patient 2 lacked a discrete Tfh population, but the size of this compartment varied markedly in healthy controls. This finding is consistent with data from the Danube cohort where average Tfh proportions were markedly lower in the patient group compared to controls, but outliers from the two groups had overlapping values [15]. This suggests that the putative Tfh defect in ICOS deficiency may be qualitative as much as quantitative; another intriguing possibility is that the size of the Tfh population could correlate with the severity of the phenotype, as has recently been reported in CD40L deficiency [19].

ICOS deficiency should be considered as part of the differential diagnosis in patients with antibody deficiency or combined immunodeficiency. An early clue is the complete lack of class switched memory B cells, which is a consistent feature of the disorder. In the absence of another molecular diagnosis we suggest that such patients should undergo an assay in which CD4^+^ T cells are activated before being evaluated for expression of ICOS and activation markers including CD69 and CD25 or HLA-DR. Inclusion of these markers is important because there is a danger that non-damaging variants in *ICOS* could be incorrectly designated as pathogenic if T cell activation is defective for another reason, resulting in a failure of ICOS induction. Evaluation of Tfh numbers might also contribute diagnostically although, as we show, these may overlap normal values.

Haematopoietic stem cell transplantation offers patients with ICOS deficiency a chance of cure should their disease be clinically severe. Although transplantation was sadly unsuccessful in our patient, one of the Kuwaiti siblings reported by Chou et al*.* was successfully transplanted with improvement in his colitis [7]. By analogy with other combined immunodeficiencies, earlier transplant is likely to be less hazardous for the patient than a later procedure, when complications have accumulated. Conservative management of patients who decline or are unsuitable for transplantation remains a difficult challenge, but we suggest a combination of immunoglobulin replacement, antimicrobial (including PJP) prophylaxis and judicious antiviral therapy.

## Electronic supplementary materials

ESM 1 (DOCX 17 kb)
