# Supplementary material for: AstuteClinician Report: A Novel 10 bp Frameshift Deletion in Exon 2 ofICOSCauses a Combined Immunodeficiency Associated with an Enteritis and Hepatitis
Source: J Clin Immunol. 2015 Sep 23;35(7):598–603. doi: 10.1007/s10875-015-0193-x (PMC4628077; doi:10.1007/s10875-015-0193-x)
Supplement: Supplementary file 1 — (DOCX 17 kb) [file 10875_2015_193_MOESM1_ESM.docx]

**A novel 10bp frameshift deletion in exon 2 of *ICOS* causes a combined immunodeficiency associated with an enteritis and hepatitis**

**Journal of Clinical Immunology**

**Authors:** Nic Robertson, Karin R Engelhardt, Neil V Morgan, Andrew J Cant, Stephen M Hughes, Mario Abinun, Yaobo Xu, Peter D Arkwright, Sophie Hambleton*^1^

*Corresponding author: sophie.hambleton@newcastle.ac.uk

(1) Institute of Cellular Medicine, Newcastle University, Newcastle upon Tyne, UK

**Supplemental Methods**

*Patients:* The parents of the two patients provided written informed consent to participate in research protocols approved by the local Research Ethics Committee (REC approval 10/H0906/022). Healthy controls donated whole blood under the same frameworks.

*Whole-exome sequencing:* Exome enrichment from DNA was performed using Illumina Truseq Exome Enrichment kit followed by sequencing using Illumina GA II Sequencing System (AROS Applied Biotechnology, Denmark). Sequences were aligned to the hg19 reference genome with BWA before realignment and variant calling by GATK. [1] Obtained variants were annotated with ANNOVAR. [2]

*Homozygosity mapping:* SNP genotyping analysis was performed using the Affymetrix Genome-Wide Human SNP Array 6.0 (AROS Applied Biotechnology) and analysed using HomozygosityMapper (http://www.homozygositymapper.org/). [3]

*Sanger sequencing:* A 390 base-pair region encompassing the putative exon 2 deletion was amplified by PCR using the forward primer AATATCCTGACATTGTCCAGCA and reverse primer CAACTGCATCTAAGTGAACTCCA. Sequences were analysed using Chromas Lite (Technelysium Pty Ltd, Australia) and Poly Peak Parser**.** [4]

*Flow cytometry:* For ICOS expression and activation assays, peripheral blood mononuclear cells (PBMC) were obtained by density gradient centrifugation over Ficoll and cryopreserved in fetal calf serum (FCS) containing 10% DMSO. Aliquots were rapidly thawed and washed into RPMI with 10% FCS, and stimulated for 18 hours at 37^o^C with PHA (5 μg/ml). Cells were then stained with Zombie Aqua Fixable Viability Kit (Biolegend) and for surface markers as indicated in the figure legends. For Tfh assays, PHA-stimulated PBMC were rested for 2 hours at 37^o^C before staining for viability and surface markers, as above. Samples were acquired using a FACSCanto II cytometer (BD Biosciences [BD]) and analysed with FlowJo (Treestar).

The following antibodies were used: CD3 PerCP (clone SK7, BD), CD4 FITC (L200, BD), CD4 PeCy7 (SK3, BD), HLA-DR FITC (L243, BD), CD69 (FN50, BD), ICOS PE (C398.4A, Biolegend) or Armenian Hamster IgG isotype control PE (HTK888, Biolegend), CXCR5 APC (51505, R&D) or IgG2B isotype control APC (13303, R&D).

**Supplementary references**

1. McKenna A, Hanna M, Banks E, Sivachenko A, Cibulskis K, et al. (2010) The Genome Analysis Toolkit: a MapReduce framework for analyzing next-generation DNA sequencing data. Genome Res 20: 1297–1303.
2. Wang K, Li M, Hakonarson H (2010) ANNOVAR: functional annotation of genetic variants from high-throughput sequencing data. Nucleic Acids Res 38: e164
3. Seelow D, Schuelke M, Hildebrandt F, Nürnberg P. (2009) HomozygosityMapper--an interactive approach to homozygosity mapping. Nucleic Acids Res.37(Web Server issue):W593-539.
4. Hill JT, Demarest BL, Bisgrove BW, Su Y-C, Smith M, et al. (2014) Poly peak parser: Method and software for identification of unknown indels using sanger sequencing of polymerase chain reaction products. Dev Dyn 243: 632–1636.
